# Altered Glutamate Homeostasis in Paramagnetic Rim Lesions of Patients with Multiple Sclerosis

**DOI:** 10.64898/2026.08.06.26359875

**Published:** 2026-08-10

**Authors:** Paul S. Jacobs, Bailey Spangler, Nikka Bakhtiar, Ahmed Elkady, Neil E. Wilson, Anshuman Swain, Elizabeth A. Horwath, Marina M. Awad, Luana Yamashita, Russell T. Shinohara, Simon Thebault, Amit Bar-Or, John A. Detre, David A. Rudko, Matthew K. Schindler, Ravinder Reddy

## Abstract

Paramagnetic rim lesions are a subset of focal white matter lesions specific to multiple sclerosis that are chronically inflamed and are associated with increased tissue injury, brain atrophy, and clinical disability. The molecular mechanisms linking paramagnetic rim lesions to these progressive biological outcomes remain unclear. Glutamatergic dysregulation has been hypothesized as a mechanism of multiple sclerosis progression potentially via excitotoxicity, but lesion-specific involvement is unknown. Here, 7T MRI was used to investigate glutamate-related metabolic alterations in paramagnetic rim lesions. Glutamate-weighted chemical exchange saturation transfer, together with T _1_ mapping and quantitative susceptibility mapping, was evaluated across paramagnetic rim lesions, non-paramagnetic rim lesions, and normal-appearing tissues in participants with multiple sclerosis (n=20) and healthy controls (n=11). Glutamate-weighted chemical exchange saturation transfer contrast was significantly higher in paramagnetic rim lesions compared to non-paramagnetic rim lesions (+10.7%) and normal-appearing white matter (+13%), while no differences were observed in normal-appearing tissue between multiple sclerosis and healthy controls. Additionally, reduced glutamate-weighted chemical exchange saturation transfer contrast in normal-appearing tissues was associated with worse motor and dexterity performance, linking observed metabolic abnormalities to clinical disability. These results identify a distinct metabolic phenotype of paramagnetic rim lesions marked by elevated glutamate-weighted signal consistent with localized excitotoxic stress. This work also implicates lesion-specific glutamatergic dysregulation in paramagnetic rim lesion-related neurodegeneration and demonstrates the potential of metabolic MRI to probe pathogenic mechanisms in multiple sclerosis.

## Full Text Availability

The license terms selected by the author(s) for this preprint version do not permit archiving in PMC. The full text is available from the preprint server.

